# Dynamic potassium variability (K-CV) as an independent predictor of mortality in maintenance hemodialysis

**DOI:** 10.1080/0886022X.2026.2664912

**Published:** 2026-09-06

**Authors:** Yeping Ma, Xiaojing Liu, Xiaoqi Wang, Li Zhongxin

**Affiliations:** Department of Nephrology, Beijing Luhe Hospital Affiliated to Capital Medical University, Beijing, China

**Keywords:** Potassium variability, hemodialysis, mortality, risk stratification, coefficient of variation, prognostic marker

## Abstract

Serum potassium fluctuations are common but understudied in maintenance hemodialysis (MHD) patients. This retrospective cohort study investigated the association between serum potassium variability, measured by the coefficient of variation (K-CV), and all-cause mortality in 201 MHD patients. Patients were divided into low and high K-CV groups according to the median K-CV cutoff and followed for a median of 72 months. Multivariate Cox regression, adjusted for confounders, revealed that patients in the high K-CV group had a significantly increased mortality risk compared to the low K-CV group (adjusted HR = 1.537, 95% CI: 1.026–2.302, *p* = 0.037). When analyzed continuously, log(K-CV) showed a strong dose-response relationship with mortality (HR = 7.627, *p* = 0.010). Subgroup analyses indicated this association was particularly pronounced in patients with lower inflammation and better nutritional status. These findings demonstrate that K-CV, a marker of serum potassium variability, serves as an independent predictor of all-cause mortality in MHD patients. Its prognostic value supports the use of K-CV for risk stratification to identify high-risk patients requiring intensive monitoring and tailored potassium management strategies in dialysis care.

## Graphical abstract

**Figure:**
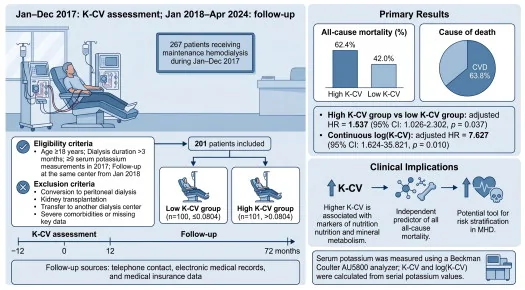

## Introduction

Maintenance hemodialysis (MHD) is one of the most commonly used renal replacement therapies for patients with end-stage kidney disease (ESKD) [1,2]. Due to the complete loss of nephron function, patients receiving MHD rely heavily on the dialysis process to regulate fluid balance, electrolyte levels, and acid-base homeostasis [3], with potassium ion (K^+^) homeostasis being particularly critical [4]. Potassium, the principal intracellular cation, plays a fundamental role in maintaining resting membrane potential, conducting neuromuscular excitation, and generating cardiac action potentials [5,6]. In recent years, increasing attention has been paid to biomarker variability. Studies have reported that blood pressure variability is significantly associated with cardiovascular events and all-cause mortality, suggesting that BP fluctuations independently predict the occurrence and progression of cardiovascular disease [7]. Similarly, serum phosphorus variability in MHD patients has attracted interest, and higher phosphorus variability is associated with increased all-cause mortality [8]. In addition, fluctuations in serum calcium levels have been linked to higher all-cause mortality in dialysis patients [9].

In the dialysis population, fluctuations in serum potassium levels are markedly greater than in patients with other chronic diseases, owing to impaired renal potassium excretion, diverse dietary habits, and the influence of multiple medications [10–12]. Previous studies have established that both hypokalemia (<3.5 mmol/L) and hyperkalemia (>5.5 mmol/L) are strongly associated with increased risks of arrhythmia, ventricular fibrillation, sudden cardiac death, and all-cause mortality, exhibiting a characteristic U-shaped relationship [13,14]. However, such studies have primarily focused on static serum potassium levels measured at a single time point, thereby overlooking the dynamic changes in potassium levels that occur throughout long-term dialysis.

Serum potassium concentration is not a static parameter but a dynamic variable influenced by multiple factors and subject to significant daily fluctuations [15]. Rapid potassium accumulation during the interdialytic period, swift clearance during dialysis sessions, and various individual factors—including diet, inflammation, metabolic status, and medication use (such as renin-angiotensin-aldosterone system inhibitors and diuretics)—can all contribute to marked short-term variations in serum potassium levels [16]. In clinical practice, it is not uncommon to observe dialysis patients whose average serum potassium levels remain within the normal range but who experience frequent and substantial fluctuations. These individuals are not necessarily at lower mortality risk [17,18]. In recent years, increasing attention has been given to the concept of biomarker variability—such as variability in blood pressure, HbA1c, and serum phosphorus—as these dynamic measures have been shown to more accurately reflect physiological instability and possess greater predictive power for clinical outcomes than static averages [19]. Accordingly, it is reasonable to hypothesize that serum potassium variability, as a quantifiable physiological fluctuation, may offer independent prognostic value beyond that of absolute potassium levels.

Preliminary evidence supports this hypothesis. For instance, a small prospective study published in 2023 by Yamaguchi et al. found that dialysis patients with a high serum potassium coefficient of variation (K-CV) had a one-year mortality rate 1.9 times higher than those in the low K-CV group, despite similar mean serum potassium levels between the two groups [17]. This finding suggests that dynamic fluctuations in potassium levels may reflect impaired homeostatic regulation, heightened metabolic stress, or deteriorating myocardial electrical stability [17]. Several retrospective studies have further reported that patients with greater potassium variability are more prone to ventricular arrhythmias, prolonged QT intervals, and sudden cardiac death [20,21]. However, these studies are limited by small sample sizes, short follow-up durations, inadequate adjustment for confounding factors, and the absence of systematic model validation. More importantly, it remains unclear whether serum potassium variability possesses independent prognostic value, whether a dose-response relationship exists, and whether this association holds consistently across different subgroups, such as stratifications by age or comorbid conditions.

In addition to insufficient evidence, there is currently no standardized method for measuring potassium variability [15]. Most studies have employed either the range (max-min) or the standard deviation (SD) to assess fluctuation magnitude. However, these measures are highly influenced by the mean and are unsuitable for comparisons among patients with differing baseline levels [22]. By contrast, the coefficient of variation (CV)—defined as the ratio of the SD to the mean—offers a normalized metric that more accurately reflects intra-individual physiological variability. CV has been widely adopted in risk assessments involving blood pressure, blood glucose, and blood lipids [23]. Moreover, the distribution of serum potassium CV is typically non-normal. Therefore, the choice of cutoff points (e.g. median or percentiles) and statistical procedures (e.g. logarithmic transformation) requires rigorous modeling and sensitivity testing [24]. Serum albumin was included as an indicator of nutritional status, although decreased albumin levels may also be influenced by systemic inflammation [25]. C-reactive protein (CRP) was considered a marker of systemic inflammation, whereas creatinine was included as a reference indicator related to muscle mass and nutritional reserve in MHD patients [26]. To further evaluate its predictive performance, survival analysis models—such as Cox regression—must be constructed, and model assumptions, including proportional hazards, multicollinearity, and goodness-of-fit, should be thoroughly examined. These critical steps have not yet been systematically addressed in earlier studies.

Current clinical practice has not yet incorporated potassium variability into the risk assessment framework for patients undergoing MHD. Monitoring of serum potassium levels remains focused on absolute abnormalities, with limited attention to variability and no established standards for its evaluation or management. This limitation may result in the underrecognition of high-risk patients and a lack of individualized intervention strategies. If serum potassium variability is confirmed to have independent prognostic significance and a quantifiable association with mortality risk, it could serve as a novel and clinically relevant dynamic biomarker. Such a marker would support decision-making in individualized management, including the optimization of dialysis prescriptions, dietary recommendations, and medication adjustments. Moreover, potassium variability is easy to measure, low in cost, and can be derived from routine laboratory tests, offering excellent feasibility and potential for widespread clinical implementation. Therefore, investigating the relationship between potassium fluctuations and patient outcomes holds substantial clinical value and health-economic implications.

Based on this rationale, the present study retrospectively analyzed 201 patients receiving MHD at a large dialysis center, examining the association between serum potassium variability—quantified by the CV over 12 months—and all-cause mortality over the subsequent six years. Kaplan-Meier survival curves, Cox proportional hazards models, Schoenfeld residual tests, and Harrell’s C-index evaluation were employed to systematically validate the statistical and clinical robustness of potassium variability as an independent predictor of mortality risk.

## Materials and methods

### Patient selection and inclusion criteria

**Figure 1.**
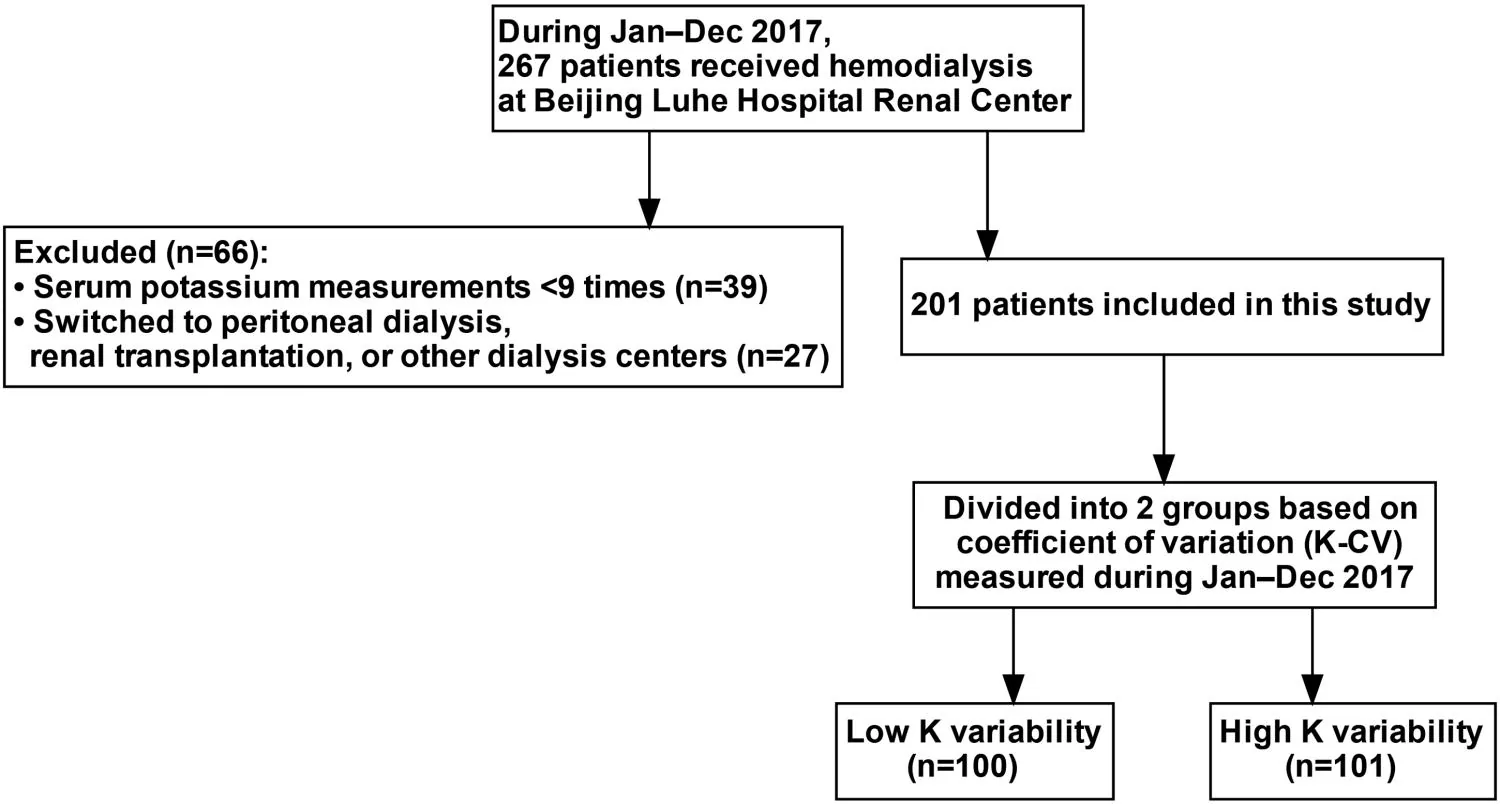
Flowchart of patient selection.

Patient data were retrieved by reviewing the hospital’s electronic dialysis system database for individuals who received MHD between January 1 and December 31, 2017. The inclusion criteria were as follows: (1) age ≥18 years; (2) diagnosis of stage 5 chronic kidney disease (CKD5); (3) dialysis duration >3 months; (4) at least nine serum potassium measurements recorded in 2017; and (5) follow-up at the same center beginning in January 2018. Exclusion criteria included: (1) conversion to peritoneal dialysis, kidney transplantation, or transfer to another dialysis center during the study period; (2) comorbid New York Heart Association (NYHA) class III-IV heart failure, active liver cirrhosis, or advanced-stage malignancy; and (3) missing data that prevented assessment of key variables. A total of 201 patients who met all eligibility criteria were included in the final analysis (Figure 1).

This study was approved by the Ethics Committee of Beijing Luhe Hospital, Capital Medical University (Approval No. 2024-LHKY-047-01). Written informed consent was obtained from all participants (or their parents/legal guardians, where applicable) in accordance with the Declaration of Helsinki.

### Serum potassium measurement and definition of variability

All serum potassium measurements were conducted by the biochemistry laboratory of our hospital. Blood samples were collected consistently in the last week of each month, on Wednesday or Thursday mornings, before dialysis sessions under non-fasting conditions. Venous blood was collected using standard vacuum tubes, promptly delivered to the laboratory, and analyzed within two hours. Serum potassium concentration was measured using the Beckman Coulter AU5800 automated biochemical analyzer *via* ion-selective electrode methodology. Internal quality control was performed daily using two-level standards, with the analytical CV maintained within 2%.

For each patient, raw serum potassium values from January to December 2017 (up to 12 measurements per individual) were extracted. The mean and SD were calculated, and the K-CV was defined as SD/mean. Given the non-normal distribution of K-CV values (Shapiro-Wilk test, *p <* 0.001), the natural logarithm of K-CV [log(K-CV)] was used in subsequent statistical models to meet the assumptions of regression analysis.

### Clinical variable collection and control of confounding factors

In January 2018, demographic and baseline clinical data were retrospectively extracted for all patients. Variables included biologic sex, age, body mass index (BMI), dialysis duration, type of primary kidney disease, comorbidities (such as hypertension, diabetes, coronary artery disease, stroke, and malignancy), dialysis prescriptions (including dialysis duration, membrane surface area, frequency, and Kt/V), and laboratory parameters (e.g. serum albumin, creatinine, and serum phosphorus). Particular attention was given to medications potentially affecting serum potassium homeostasis, including angiotensin-converting enzyme inhibitors (ACEIs), angiotensin II receptor blockers (ARBs), spironolactone, and triamterene. These medications were incorporated into the multivariable models as covariates and analyzed through subgroup stratification in sensitivity analyses.

To minimize confounding from severe baseline conditions, patients with end-stage heart failure, decompensated liver function, or advanced malignancies were excluded during the study design phase. The overall rate of missing data for variables was less than 5%. No imputation was performed, and complete-case analysis was adopted.

### Definition of endpoints and follow-up methods

The primary endpoint was all-cause mortality, defined as death from any cause occurring between January 1, 2018, and April 30, 2024. The secondary endpoint was cardiovascular mortality, which included deaths due to myocardial infarction, fatal arrhythmias, acute heart failure, stroke, peripheral vascular events, and sudden death of unknown cause. All mortality data were verified through a triad of sources: outpatient/inpatient medical records, telephone follow-ups with family members, and health insurance deregistration information to ensure accuracy. Patients who were lost to follow-up or relocated were treated as right-censored in the survival analysis.

### Sample size estimation

Sample size estimation was conducted using PASS 15.0 software based on the statistical framework of the Cox proportional hazards model. According to prior studies, the all-cause mortality rate in the MHD population over a 5–6 year follow-up period is approximately 50%. Assuming a hazard ratio (HR) of 1.5 for K-CV and setting a two-sided significance level (*α*) of 0.05 with 80% power (1–*β*), the minimum required sample size was calculated to be 174 patients. To account for potential data loss due to dropout, referral, or follow-up attrition, an additional 10–15% was added as a redundancy margin, resulting in a target sample size of 200. Ultimately, 201 patients were included in the final analysis, satisfying the theoretical sample size requirements and ensuring adequate statistical power and robustness of the findings.

### Statistical analysis

All statistical analyses were performed using SPSS version 22.0 (IBM Corp., Armonk, NY, USA) and R software version 4.2.2 (R Foundation for Statistical Computing, Vienna, Austria). Continuous variables with a normal distribution were expressed as mean ± SD, while those with a non-normal distribution were presented as median and interquartile range (IQR). Categorical variables were reported as frequencies and percentages. Comparisons of continuous variables between two groups were conducted using the independent samples t-test or the Mann-Whitney *U* test, depending on the distribution. Categorical variables were compared using the chi-square (*χ*^2^) test or Fisher’s exact test, as appropriate. The correlation between the K-CV and other clinical indicators was assessed using Pearson or Spearman correlation analysis, depending on variable distribution.

Survival analysis was conducted using the Kaplan-Meier method to estimate survival curves, with group differences assessed by the log-rank test. Cox proportional hazards models were applied to evaluate the association between K-CV and both all-cause and cardiovascular mortality, including both univariable and multivariable models. The multivariable models were adjusted for potential confounders, including age, serum albumin, creatinine, single-pool Kt/V (spKt/V), serum phosphorus, serum sodium, CRP, and the use of potassium-sparing agents. Interaction terms were included in subgroup analyses to assess effect modification across stratified variables. All tests were two-tailed, and a *p*-value < 0.05 was considered statistically significant. The proportional hazards assumption was assessed using Schoenfeld residuals; no covariates showed significant violations (*p* > 0.05), as presented in the (supplementary table Table S1).

## Results

### Baseline characteristics were generally balanced between high and low serum potassium variability groups

A total of 201 MHD patients were included in the study, with 100 patients assigned to the low potassium variability group (K-CV ≤ 0.0804) and 101 to the high variability group (K-CV > 0.0804). No significant differences were observed between the two groups in sex distribution (male: 61.0 vs. 57.4%, *p =* 0.606), dialysis duration (57 vs. 51 months, *p =* 0.323), or BMI (23.2 ± 3.7 vs. 23.5 ± 3.4 kg/m^2^, *p =* 0.608). However, patients in the high variability group were older on average (61.5 ± 12.9 vs. 57.7 ± 13.1 years, *p =* 0.037). There were no statistically significant differences in the distribution of primary kidney disease or major comorbidities between the two groups, although the proportions of diabetic nephropathy, coronary artery disease, and cancer were slightly higher in the high variability group (*p =* 0.101, 0.060, and 0.054, respectively). Regarding laboratory indicators, the high variability group had lower serum albumin levels (4.16 ± 0.37 vs. 4.25 ± 0.29 g/dL, *p =* 0.024) and higher intact parathyroid hormone (iPTH) levels (234.6 vs. 145.9 pg/mL, *p =* 0.007). Other parameters, including mean serum potassium, serum phosphorus, calcium, CRP, and Kt/V, showed no significant differences between groups (all *p >* 0.05, Table 1). In summary, aside from differences in age, albumin, and iPTH, the two groups were generally well balanced in terms of demographic and laboratory characteristics. These differences were adjusted for in the multivariable analysis.

**Table 1.** Patient characteristics overall and by serum potassium variability.

	Standard reference range	All (*N* = 201)	Low K variability (*n* = 100)	High K variability (*n* = 101)	*p* value
Age (years)		59.6 ± 13.1	57.7 ± 13.1	61.5 ± 12.9	0.037
Males (*n*, %)		119 (59.2)	61 (61.0)	58 (57.4)	0.606
Duration of dialysisa (months)		56 (34–86)	57 (41–96)	51 (32–79)	0.323
BMI (kg/m^2^)	18.5–24.9	23.4 ± 3.5	23.2 ± 3.7	23.5 ± 3.4	0.608
Primary kidney diseases (*n*, %)					0.101
Glomerulonephritis (*n*, %)		66 (32.8)	39 (39.0)	27 (26.7)	
Diabetic nephropathy (*n*, %)		61 (30.3)	23 (23.0)	38 (37.6)	
Hypertensive nephropathy (*n*, %)		27 (13.4)	15 (15.0)	12 (11.9)	
Others (*n*, %)		47 (23.4)	23 (23.0)	24 (23.8)	
Comorbidities (*n*, %)					
Hypertension (*n*, %)		174 (86.6)	85 (85.0)	89 (88.1)	0.813
Diabetes (*n*, %)		76 (37.8)	33 (33.0)	43 (42.6)	0.162
Coronary heart disease (*n*, %)		57 (28.4)	22 (22.0)	35 (34.7)	0.060
Cerebrovascular diseases (*n*, %)		31 (15.4)	14 (14.0)	17 (16.8)	0.578
Cancer (*n*, %)		10 (5.0)	2 (2.0)	8 (7.9)	0.054
Systolic blood pressure (mmHg)	90–140	147 ± 16	147 ± 15	147 ± 18	0.814
Diastolic blood pressure (mmHg)	60–90	78 ± 11	78 ± 11	77 ± 11	0.389
Hemoglobin (g/dL)	12.0–16.0 (male)； 11.0–15.0 (female)	11.63 ± 1.15	11.59 ± 1.01	11.66 ± 1.28	0.649
White blood cell (*10^9^/L)	4.0–11.0	6.0 (4.9–7.1)	6.0 (4.9–7.0)	6.0 (5.1–7.2)	0.866
Neutrophils (*10^9^/L)	1.8–7.5	4.0 (3.2–5.0)	3.9 (3.3–4.9)	4.0 (3.2–5.1)	0.763
Platelets (*10^9^/L)	100–300	173.4 ± 51.3	172.9 ± 50.2	174..4 ± 52.6	0.842
Albumin (g/dL)	3.5–5.0	4.21 ± 0.34	4.25 ± 0.29	4.16 ± 0.37	0.024
C-reactive protein (mg/L)	<10	2.7 (1.4–7.9)	2.8 (1.4–6.1)	2.6 (1.3–10.5)	0.664
Mean serum Potassium (mmol/L)	3.5–5.0	4.84 ± 0.67	4.85 ± 0.65	4.83 ± 0.69	0.884
Sodium (mmol/L)	135–145	138.2 ± 2.8	138.4 ± 2.5	138.1 ± 3.0	0.388
Chloride (mmol/L)	96–106	99.7 ± 3.27	99.8 ± 3.2	99.6 ± 3.3	0.693
Calcium (mmol/L)	2.1–2.6	2.2 (2.1–2.3)	2.2 (2.0–2.3)	2.2 (2.1–2.4)	0.457
Phosphorus (mmol/L)	0.8–1,045	1.7 ± 0.5	1.7 ± 0.5	1.7 ± 0.5	0.592
Intact parathyroid hormone (pg/mL)	15–65	201.3 (112.7–343.5)	234.6 (121.1–378.5)	145.9 (94.5–261.8)	0.007
Creatinine (mg/dL)	0.6–1.2 (male); 0.5–1.1 (female)	9.82 ± 2.71	9.99 ± 2.72	9.64 ± 2.70	0.365
Blood urea nitrogen (mmol/L)	3.2–7.1	22.7 ± 5.3	23.0 ± 5.0	22.5 ± 5.7	0.439
Total cholesterol (mmol/L)	3.1–5.2	4.3 ± 1.1	4.3 ± 1.1	4.2 ± 1.1	0.597
Triglyceride (mmol/L)	0.56–1.7	1.5 (1.0–2.3)	1.6 (1.0–2.2)	1.4 (1.0–2.4)	0.885
Plasma bicarbonate (mmol/L)	22–28	23.0 ± 2.8	23.0 ± 2.7	23.1 ± 2.9	0.645
spKt/V	>1.2	1.35 ± 0.31	1.36 ± 0.34	1.34 ± 0.29	0.763
Interdialytic weight gain (kg)		2.32 ± 0.91	2.28 ± 0.92	2.38 ± 0.89	0.428
Fistula use (*n*, %)		192 (95.5)	95 (95.0)	97 (96.0)	0.840
All-cause mortality (*n*, %)		105 (52.2)	42 (42.0)	63 (62.4)	0.004
Cardiovascular mortality (*n*, %)		67 (33.3)	27 (27.0)	40 (39.6)	0.058

Note: Abbreviations: BMI: body mass index; spKt/V: single-pooled Kt/V.

### Distribution of deaths and causes of mortality

**Figure 2.**
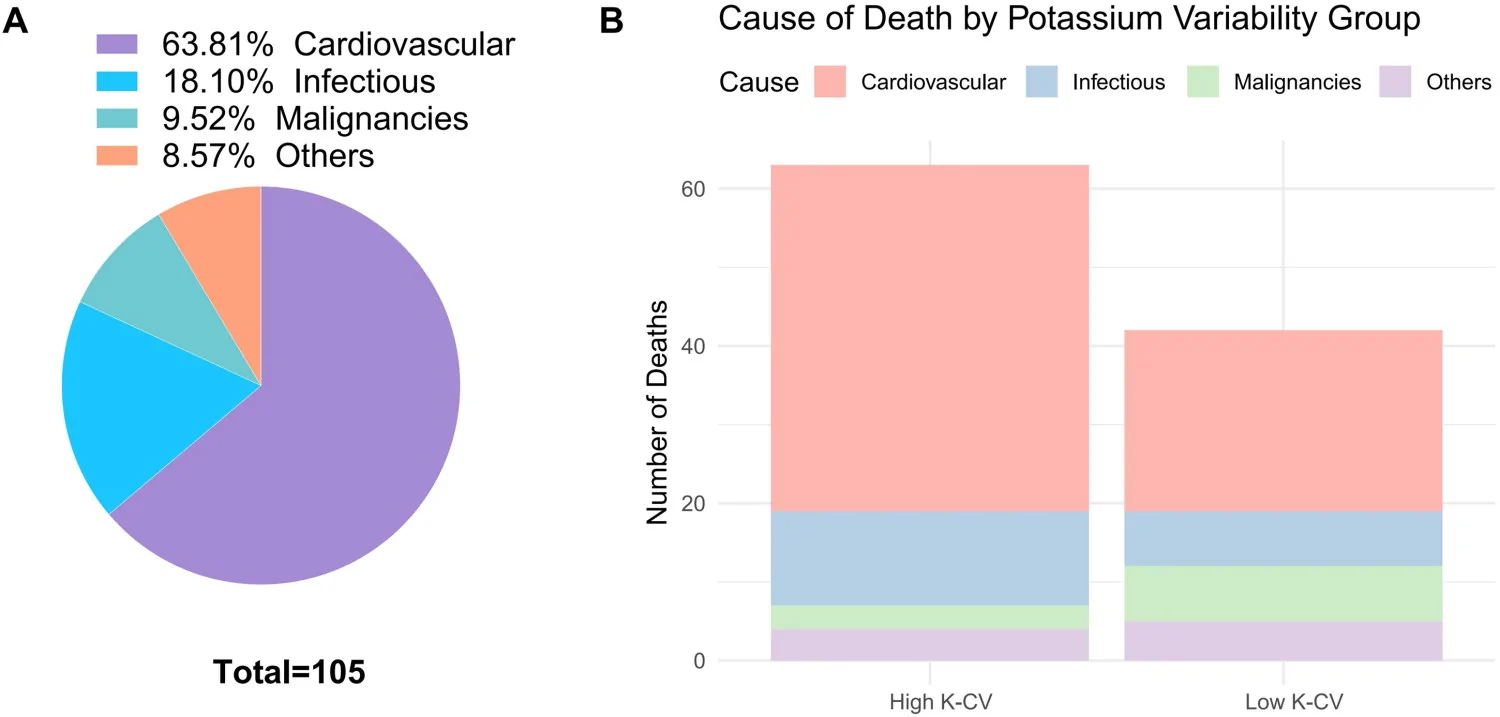
Distribution of causes of death across serum potassium variability groups. Note: (A) Pie chart illustrating the causes of death among 105 deceased patients; cardiovascular events accounted for the largest proportion at 63.8%. (B) Bar chart displaying the distribution of causes of death between the high variability group and the low variability group based on serum potassium variability.

The all-cause mortality rate was higher in the high potassium variability group, although the distribution of causes of death was similar to that in the low variability group. During a median follow-up of 72 months (IQR: 66–76 months), a total of 105 deaths were recorded, yielding an overall mortality rate of 52.2%. Specifically, 63 deaths occurred in the high variability group (62.4%) and 42 in the low variability group (42.0%). Analysis of cause-specific mortality revealed that cardiovascular events were the leading cause of death, accounting for 63.8% of all deaths, with comparable proportions between groups (63.5% vs. 64.3%, *p =* 0.934). Other causes included infections (18.1%), malignancies (9.5%), and miscellaneous conditions (8.6%), with no statistically significant differences observed between groups (all *p >* 0.05; see Figure 2 and Table 2). These findings suggest that serum potassium variability exerts its influence primarily through elevating overall mortality risk rather than altering the distribution of specific causes of death.

**Table 2.** Causes of death.

Causes of deaths (*n*, %)	All (*n* = 105)	Low K variability (*n* = 42)	High K variability (*n* = 63)	*p* value
Cardiovascular	67 (63.8)	27 (64.3)	40 (63.5)	0.934
Infectious	19 (18.1)	7 (16.7)	12 (19.0)	0.756
Malignancies	10 (9.5)	3 (7.1)	7 (11.1)	0.497
Others	9 (8.6)	5 (11.9)	4 (6.4)	0.319

### Serum potassium variability was associated with several clinical parameters

**Figure 3.**
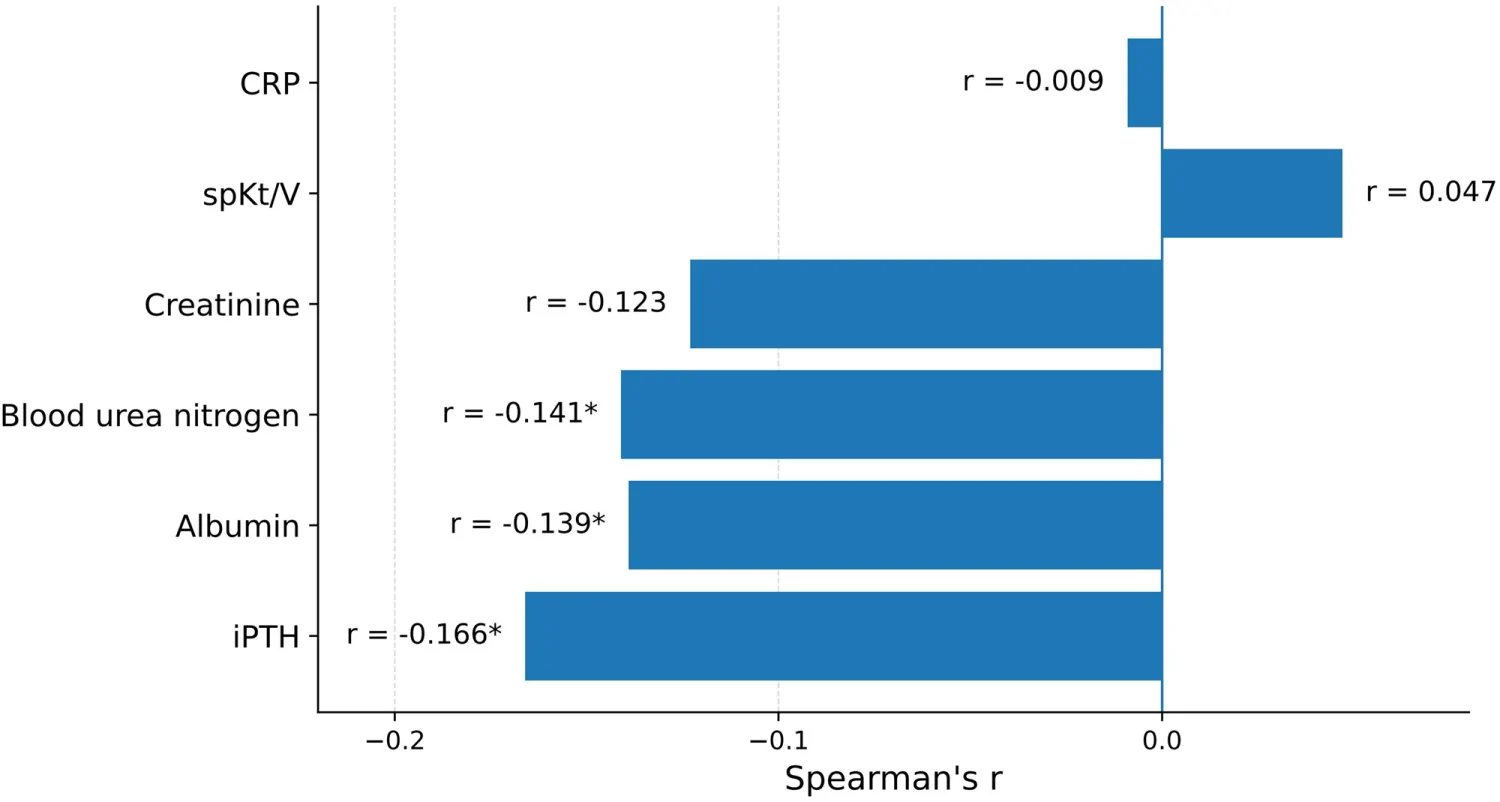
Correlation analysis between K-CV and selected clinical parameters. Note: Spearman’s rank correlation coefficients were used to assess the association between K-CV and selected clinical indicators, including albumin, iPTH, blood urea nitrogen, creatinine, CRP, and spKt/V. Bar lengths represent the strength of correlation, with direction indicating positive or negative correlation. Asterisks (*) denote statistically significant correlations* (p < 0.05)*.

To investigate the clinical characteristics associated with elevated K-CV, correlations between K-CV and multiple indicators were analyzed (Table 3, Figure 3). The results demonstrated a significant negative correlation between K-CV and serum albumin (*r* = −0.139, *p =* 0.039), iPTH (*r* = −0.166, *p =* 0.021), and blood urea nitrogen (*r* = −0.141, *p =* 0.046). No significant correlations were observed between K-CV and creatinine, mean serum potassium, CRP, or Kt/V (all *p >* 0.05). These findings suggest that serum potassium variability is associated with nutritional and mineral metabolism-related markers, rather than with creatinine or CRP (see Table 3 for details).

**Table 3.** Variables correlation with serum potassium variability.

Variables	*r*	*p* value
Age	0.134	0.037
Sex	0.108	0.127
Dialysis vintage (year)	−0.060	0.4
BMI	0.056	0.432
Diabetes	0.136	0.055
Systolic blood pressure (mmHg)	0.070	0.326
Diastolic blood pressure (mmHg)	−0.050	0.480
Hemoglobin (g/dL)	0.049	0.487
Albumin (g/dL)	−0.139	0.039
C-reactive protein (mg/L)	−0.009	0.903
Mean serum Potassium (mmol/L)	0.129	0.068
Calcium (mmol/L)	0.057	0.424
Phosphorus (mmol/L)	−0.064	0.372
Intact parathyroid hormone (pg/mL)	−0.166	0.021
Creatinine (mg/dL)	−0.123	0.082
Blood urea nitrogen (mmol/L)	−0.141	0.046
Plasma bicarbonate (mmol/L)	0.086	0.232
spKt/V	0.047	0.504

### All-cause mortality risk was significantly higher in the high potassium variability group, and K-CV served as an independent adverse prognostic factor

**Figure 4.**
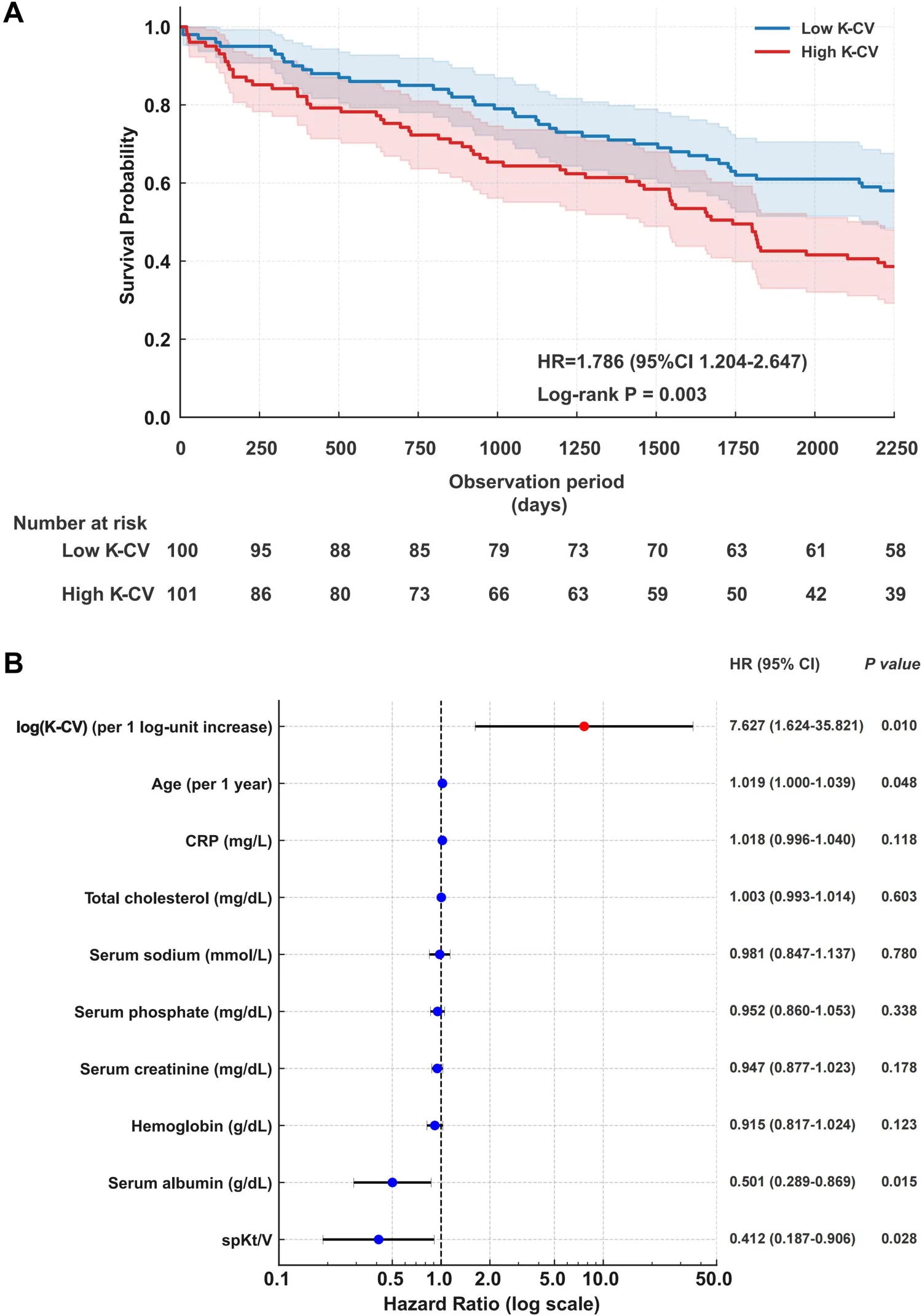
Kaplan-Meier survival curves and multivariable Cox regression forest plot. Note: (A) Kaplan-Meier survival curves comparing patients in the high and low potassium variability groups (*n* = 201) during the follow-up period. Time is expressed in days. The shaded areas represent the 95% CIs, and the table below indicates the number at risk at each time point. (B) Forest plot derived from a multivariable Cox proportional hazards model, illustrating the HR and 95% CI for all-cause mortality associated with the K-CV (modeled as log(K-CV) and covariates. The x-axis uses a logarithmic scale.

In the Kaplan-Meier analysis, the cumulative survival rate in the high potassium variability group was significantly lower than that in the low variability group (log-rank test, *p =* 0.003) (Figure 4A). In the Cox proportional hazards model, the high variability group showed a significantly elevated risk of all-cause mortality compared to the low variability group (unadjusted model: H *r* = 1.786, 95% confidence interval [CI]: 1.204–2.647, *p =* 0.004; multivariable-adjusted model: H *r* = 1.537, 95% CI: 1.026–2.302, *p =* 0.037) (Table 4).

**Table 4.** Relative risk for death in the high K variability group compared to the low K variability group.

K-CV (categorical)	Crude HR (95% CI)	*p* value	Adjusted HR (95% CI)	*p* value
Low K-CV (≤0.0804)	Reference	–	Reference	–
High K-CV (>0.0804)	1.786 (1.204–2.647)	0.004	1.537 (1.026–2.302)	0.037

Note: Adjusted for age, serum albumin, serum creatinine, Kt/V, and other potential confounders.

When log(K-CV) entered into the model as a continuous variable, each 1-unit increase in log(K-CV) was associated with a significantly increased risk of all-cause mortality (unadjusted model: H *r* = 8.303, 95% CI: 1.936–36.605, *p =* 0.004; multivariable-adjusted model: H *r* = 7.627, 95% CI: 1.624–35.821, *p =* 0.010). Here, the HR represents the risk associated with each 1-unit increase in log(K-CV), which corresponds to an e-fold (≈2.718-fold) relative increase in K-CV (Table 5, Figure 4B). For clinical interpretability, this is approximately equivalent to an HR of 1.21 for each 10% relative increase in K-CV and 4.09 for each doubling of K-CV.

**Table 5.** Cox proportional hazards regression model for all-cause of mortality in MHD patients.

	Univariate		Multivariate	
	Crude HR (95% CI)	*p* value	Adjusted HR (95% CI)	*p* value
Log(K-CV) per 1 unit	8.303 (1.936–36.605)	0.004	7.627 (1.624–35.821)	0.01
Mean K per 1 unit	0.659 (0.454–0.955)	0.027	1.104 (0.710–1.718)	0.660
Age/year	1.073 (1.053–1.094)	<0.001	1.066 (1.042–1.090)	<0.001
Female vs. male	0.818 (0.550–1.215)	0.320		
Dialysis vintage/year	0.977 (0.926–1.030)	0.384		
Diabetes history	1.980 (1.348–2.908)	<0.001	1.057 (0.649–1.720)	0.824
Coronary heart disease	2.204 (1.485–3.272)	<0.001	1.013 (0.606–1.692)	0.790
Hemoglobin per 1 unit	0.780 (0.651–0.933)	0.007	1.127 (0.606–1.670)	0.331
Albumin per 1 unit	0.375 (0.292–0.482)	<0.001	0.321 (0.158–0.650)	0.002
Creatinine per 1 unit	0.808 (0.748–0.873)	<0.001	0.907 (0.827–0.994)	0.040
TC per 1 unit	0.780 (0.636–0.955)	0.016	0.919 (0.724–1.168)	0.491
Na per 1 unit	0.882 (0.818–0.951)	0.001	0.993 (0.906–1.088)	0.876
Phosphate per 1 unit	0.615 (0.408–0.928)	0.020	1.039 (0.592–1.822)	0.894
Plasma bicarbonate per 1 unit	0.919 (0.858–0.985)	0.018	1.023 (0.931–1.125)	0.636
CRP per 1 unit	1.022 (1.014–1.030)	<0.001	1.003 (0.992–1.013)	0.611
KTV per 1 unit	0.228 (0.127–0.410)	<0.001	0.263 (0.137–0.504)	<0.001

Note: Adjusted for age, serum albumin, serum creatinine, Kt/V, and other potential confounders.

The multivariable model was adjusted for age, albumin, creatinine, spKt/V, CRP, serum sodium, serum phosphorus, and total cholesterol. To assess the incremental predictive value of log(K-CV), we compared model discrimination and fit with and without log(K-CV). After adding log(K-CV), Harrell’s C-index increased from 0.701 to 0.734, and AIC decreased from 828.4 to 817.2, indicating improved discrimination and model performance (Table S2). Discrimination and reclassification also improved significantly (IDI = 0.026, *p* = 0.018; NRI = 0.142, *p* = 0.022), supporting the incremental prognostic value of K-CV beyond standard predictors.

### Subgroup analysis revealed variability in the association between K-CV and mortality risk across different clinical conditions

**Figure 5.**
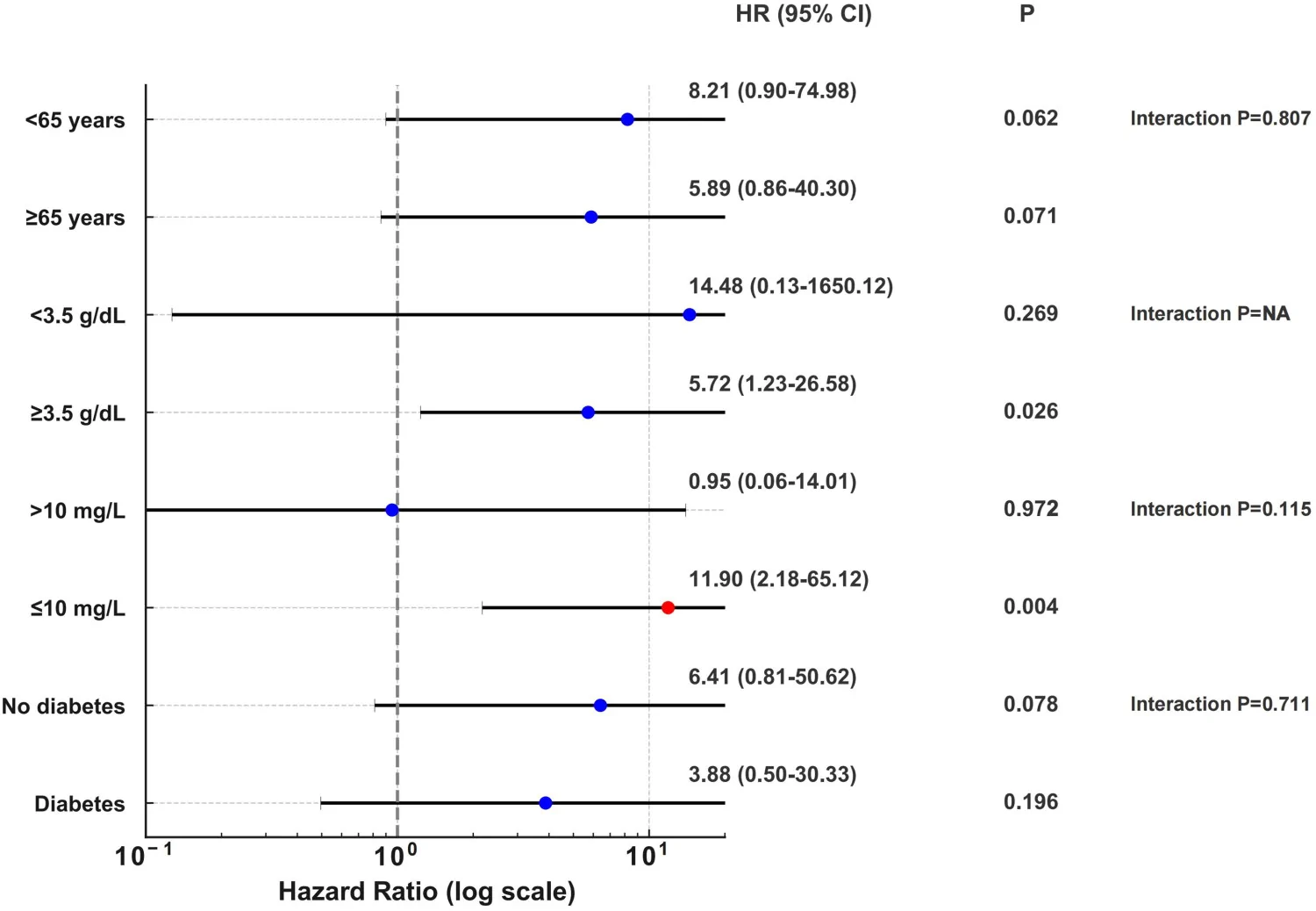
Association between log(K-CV) and all-cause mortality across subgroups. Note: Forest plot showing the HRs and 95% CIs for all-cause mortality associated with log(K-CV) across different subgroups, including age (<65 vs ≥65 years), albumin (<3.5 vs ≥3.5 g/dL), CRP (>10 vs ≤10 mg/L), and diabetes status (absent vs present). *p*-values for within-group comparisons and interaction terms are listed on the right. The dashed vertical line represents the null value (HR = 1). Confidence intervals exceeding the plotting range were truncated for visualization.

To evaluate the impact of K-CV on mortality risk under different clinical conditions, subgroup analyses were conducted based on age, nutritional status (serum albumin), and inflammatory status (CRP) (Figure 5, Table 6). In the age-stratified analysis, log(K-CV) was positively associated with mortality risk in both the <65 years and ≥65 years subgroups (H *r* = 8.21, 95% CI: 0.90–74.98, *p =* 0.062; H *r* = 5.89, 95% CI: 0.86–40.30, *p =* 0.071), although these associations did not reach statistical significance. In the nutritional status stratification, log(K-CV) was a significant predictor of mortality in patients with serum albumin ≥3.5 g/dL (H *r* = 5.72, 95% CI: 1.23–26.58, *p =* 0.026). In contrast, although the HR was higher in the low albumin subgroup, the association was not statistically significant (H *r* = 14.48, 95% CI: 0.13–1650.12, *p =* 0.269), potentially due to limited sample size. Inflammation-based stratification showed that log(K-CV) was strongly associated with mortality risk in the low CRP group (≤10 mg/L) (H *r* = 11.90, 95% CI: 2.18–65.12, *p =* 0.004), whereas no significant association was observed in the high CRP group (H *r* = 0.95, *p =* 0.972). No significant differences were found in the diabetes-stratified subgroup.

**Table 6.** Subgroup analysis of the association between log(K-CV) and all-cause mortality.

Subgroup	Level	HR (95% CI)	*p* value	Interaction *p* value
Age	<65 years	8.21 (0.90–74.98)	0.062	0.807
	≥65 years	5.89 (0.86–40.30)	0.071	
Albumin	<3.5 g/dL	14.48 (0.13–1650.12)	0.269	0.116
	≥3.5 g/dL	5.72 (1.23–26.58)	0.026*	
CRP	>10 mg/L	0.95 (0.06–14.01)	0.972	0.116
	≤10 mg/L	11.90 (2.18–65.12)	0.042*	
Diabetes	No	6.41 (0.81–50.62)	0.078	0.711
	Yes	3.88 (0.50–30.33)	0.196	

Note: Values are hazard ratios (HR) with 95% confidence intervals (CI). Interaction P values were derived from models including the cross-product of log(K-CV) and subgroup variables.

Although none of the interaction terms reached statistical significance, the observed trends suggest that the impact of K-CV on mortality risk may be more pronounced in patients with lower inflammation and better nutritional status, indicating a greater sensitivity to serum potassium fluctuations in these populations.

## Discussion

This study evaluated the association between serum potassium variability (K-CV) and risks of all-cause and cardiovascular mortality in a MHDcohort. Higher K-CV was significantly associated with increased mortality risk and remained independently associated after multivariable adjustment, highlighting that potassium fluctuations, beyond a single potassium value, provide additional long-term prognostic information. The median follow-up of 72 months offers a robust basis for assessing long-term outcomes.

Evidence linking potassium variability to adverse outcomes in MHD has been observed across different study designs. At the patient level, higher intra-individual potassium coefficient of variation has been independently associated with mortality [15]. At the facility level, multicenter analyses assessing within-center dispersion of potassium have reported higher facility-level variability to be associated with greater risks of all-cause and cardiovascular death [27]. Similar patterns have been reported in other chronic diseases: HbA1c variability is associated with all-cause mortality in type 2 diabetes [28], and blood pressure variability predicts cardiovascular events independent of mean blood pressure [29], suggesting that variability may capture impaired homeostatic stability beyond average levels. Notably, facility-level “dispersion” may largely reflect practice patterns and case-mix differences and is not directly actionable for individual risk stratification, whereas patient-level evidence remains relatively limited regarding follow-up duration, heterogeneity of effects, and model performance evaluation [15,18]. In this context, we derived patient-level K-CV from routinely collected pre-dialysis potassium measurements, modeled K-CV using both categorical grouping and log(K-CV) as a continuous exposure to characterize a dose–response relationship, assessed its incremental prognostic value beyond conventional risk factors, and examined effect heterogeneity across clinically relevant subgroups, thereby providing a more complete evidence chain for incorporating variability metrics into individualized risk assessment.

Most prior studies on serum potassium have focused on “static” absolute levels (hyperkalemia or hypokalemia) and their associations with mortality, particularly in relation to acute arrhythmias and cardiovascular events [13,30]. Large observational studies, including DOPPS, have reported associations between static hyperkalemia and mortality [31,32], and several studies continue to use absolute potassium values as the primary exposure [33,34]. However, single measurements or mean values may not adequately reflect chronic risk conveyed by long-term fluctuations. Accordingly, we used the coefficient of variation to quantify potassium variability and applied log transformation to improve interpretability and suitability for continuous modeling, thereby capturing long-term risk information not apparent from conventional point estimates.

**Figure 6.**
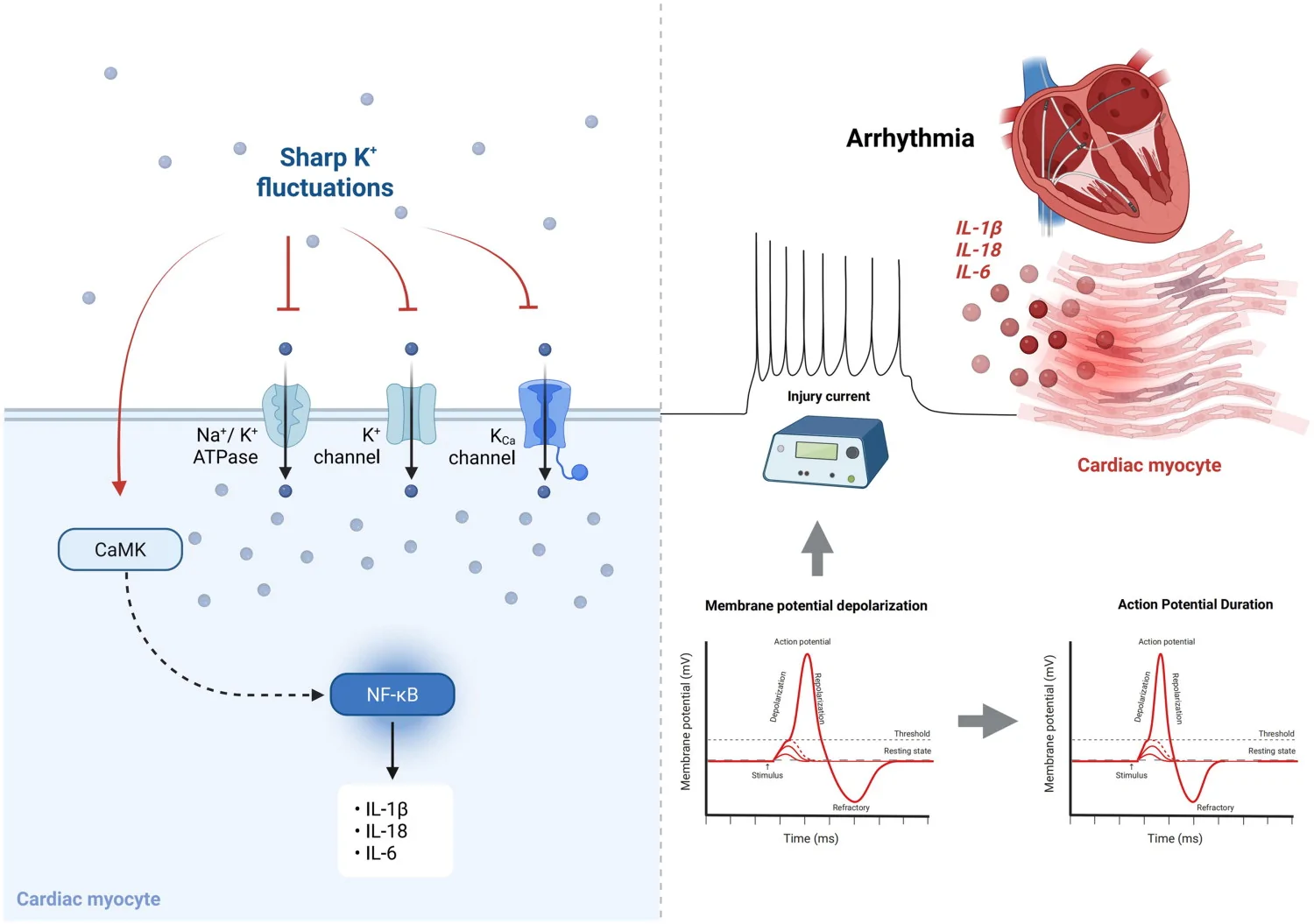
Proposed pathway linking potassium fluctuations to mortality in maintenance hemodialysis.

Mechanistically, marked pre- to post-dialysis potassium shifts may trigger malignant ventricular arrhythmias; potassium-driven arrhythmogenesis is not solely a function of a single “high” or “low” value and may contribute to sudden death [35]. Substantial potassium transfer during hemodialysis has also been linked to autonomic dysfunction [36]. In addition, abnormal potassium concentrations can destabilize cardiac electrophysiology through multiple pathways, including altered K^+^ channel conductance, inhibition of Na^+/^K^+^-ATPase activity, activation of CaMK signaling, membrane depolarization, shortening of action potential duration, and generation of injury currents, collectively increasing arrhythmia risk [37]. These mechanisms support a pathway whereby interdialytic potassium variability increases repolarization heterogeneity, promotes arrhythmogenesis, and contributes to mortality (Figure 6).

Beyond electrophysiology, elevated K-CV may reflect broader systemic conditions, including nutritional status, inflammation, and dialysis adequacy [38,39]. In our cohort, patients with higher K-CV had lower albumin and higher iPTH levels, suggesting links to malnutrition and secondary hyperparathyroidism. Subgroup analyses further indicated stronger associations in patients with lower inflammatory burden (CRP ≤10 mg/L) and better nutritional status (albumin ≥3.5 g/dL), implying that when background disturbances are less prominent, K-CV may better capture intrinsic metabolic instability associated with mortality, thereby supporting more refined individualized risk assessment.

Conceptually, the “biological variability paradigm” posits that greater fluctuations in physiological parameters reflect diminished homeostatic reserve and resilience, serving as an early signal of system vulnerability beyond single absolute values [40]. Our findings extend this paradigm to electrolyte management and support serum potassium variability (K-CV) as a dynamic warning marker that complements conventional risk assessment. Given international differences in diet, potassium sources, dialysis prescriptions, and adherence, external validation in diverse populations remains necessary to establish generalizability and implementation potential.

The novelty of this study lies in the following: (1) the long follow-up (median 72 months), enabling a more reliable assessment of long-term mortality risk; (2) the focus on potassium variability as a dynamic phenotype, quantified by K-CV and log(K-CV) and characterized using both categorical and continuous modeling, demonstrating a significant association with all-cause mortality; (3) refined subgroup analyses showing stronger signals in specific clinical phenotypes (lower inflammation and better nutrition), supporting individualized risk stratification; and (4) the high translational potential of K-CV, which can be derived from routine laboratory testing and may serve as a practical complement to standard risk assessment.

Several limitations should be acknowledged, together with directions for future work. (1) This single-center retrospective design may introduce selection bias; some subgroups were small with limited power and wider confidence intervals, and the findings require confirmation in large prospective studies. (2) Although we adjusted for key determinants such as medication use and dialysis prescription and documented RAAS inhibitor use, several important drivers were not fully quantified, including dietary potassium intake, interdialytic weight gain, potassium binder use, and interdialytic fluid and sodium intake, limiting a comprehensive understanding of determinants and modifiable targets. (3) More systematic capture of dialysate composition and detailed prescription parameters is needed to clarify their relationships with potassium variability and to inform feasible prescription/dialysate optimization strategies. (4) Geographic and ethnic heterogeneity is plausible given cross-regional differences in diet, potassium sources, prescriptions, and adherence, underscoring the need for external validation. (5) Future multicenter prospective cohorts with standardized data collection should integrate dietary and interdialytic behavioral data, potassium binder use, dialysate composition, detailed prescription parameters, and granular medication exposure (dose, duration, combinations) to better define determinants and test interventions aimed at reducing potassium variability and improving the reliability of risk prediction.

Because serum potassium is routinely measured in hemodialysis care, K-CV (SD/mean) can be automatically calculated from laboratory feeds and displayed in EMR dashboards alongside mean potassium and recent trends; in practice, a rolling window (e.g. the prior 6–12 months) can be updated with each new pre-dialysis result. For risk stratification, “high variability” can be defined using our study cutoff (K-CV > 0.0804), with an optional “very high variability” tier based on center-specific percentiles (e.g. top quartile) to accommodate differences in testing frequency and practice patterns and the known measurement variability in potassium interpretation [41]. When persistently elevated K-CV is detected, the EMR could prompt standardized, actionable reviews (dialysate potassium prescription, dietary counseling, and medication reconciliation) while limiting alert fatigue by requiring elevation across consecutive measurements. Beyond rule-based alerts, K-CV could also be integrated into AI-enabled dialysis analytics or remote monitoring workflows to support earlier identification of unstable potassium control and timely intervention between visits [42–44].

## Conclusion

This study systematically evaluated the association between K-CV and the risk of all-cause mortality in patients undergoing MHD. The results demonstrated that elevated K-CV was significantly associated with increased mortality risk and retained independent prognostic value after adjusting for multiple confounding factors, underscoring its potential as a prognostic biomarker. The inclusion of log(K-CV) as a continuous variable further enhanced the robustness of the findings, revealing that each unit increase in log(K-CV) was significantly correlated with higher mortality risk, indicating a clear dose-response relationship and strong clinical interpretability. Moreover, subgroup analyses showed that the predictive value of K-CV was more pronounced among individuals with lower levels of inflammation (CRP ≤10 mg/L) and better nutritional status (albumin ≥3.5 g/dL), suggesting that K-CV may serve as a more sensitive indicator of underlying health fluctuations in these populations. These findings indicate that K-CV reflects not only biochemical instability but may also represent an integrated marker of nutritional and inflammatory status contributing to long-term prognosis. In conclusion, serum potassium variability, as a convenient, dynamic, and quantifiable parameter, holds promise for incorporation into routine risk stratification and monitoring strategies in dialysis patients, particularly as an early warning indicator in low-risk phenotypic groups.

## Supplementary Material

Revised_Table_S1_and_S2 new.docx

## Data Availability

All data generated or analyzed during this study are included in this article. Further enquiries can be directed to the corresponding author.

## Abbreviations

BMI: Body Mass Index
CI: Confidence Interval
CRP: C-Reactive Protein
CV: Coefficient of Variation
HR: Hazard Ratio
IQR: Interquartile Range
iPTH: Intact Parathyroid Hormone
K-CV: Serum Potassium Coefficient of Variation
log(K-CV): Natural Logarithm of the Serum Potassium Coefficient of Variation
MHD: Maintenance Hemodialysis
SD: Standard Deviation
spKt/V: Single-Pool Kt/V

## References

[1] Beers KH, Sperati CJ, Weisman DS, et al. Improving primary care delivery for patients receiving maintenance hemodialysis. Am J Kidney Dis. 2021;78(6):886–891. doi: 10.1053/j.ajkd.2021.02.340. 33992728

[2] Kim HW, Jhee JH, Joo YS, et al. Clinical significance of hemodialysis quality of care indicators in very elderly patients with end stage kidney disease. J Nephrol. 2022;35(9):2351–2361. doi: 10.1007/s40620-022-01356-3. 35666374

[3] El-Housseini Y, Mahfoudh H, Jarraya F, et al. Transpiration stimulée afin d’améliorer l’équilibre hydro-sodé chez les patients hémodialysés: à propos d’un cas. Néphrol Thér. 2012;8(6):472–475. doi: 10.1016/j.nephro.2012.02.001. 22537514

[4] Sharma MK, Wieringa FP, Frijns AJH, et al. On-line monitoring of electrolytes in hemodialysis: on the road towards individualizing treatment. Expert Rev Med Devices. 2016;13(10):933–943. doi: 10.1080/17434440.2016.1230494. 27653043

[5] Tricarico D, Barbieri M, Camerino DC. Taurine blocks ATP-sensitive potassium channels of rat skeletal muscle fibres interfering with the sulphonylurea receptor. Br J Pharmacol. 2000;130(4):827–834. doi: 10.1038/sj.bjp.0703385. 10864889 PMC1572140

[6] Ohyama H, Kajita H, Omori K, et al. Inhibition of cardiac delayed rectifier K + currents by an antisense oligodeoxynucleotide against IsK (minK) and over-expression of IsK mutant D77N in neonatal mouse hearts. Pflugers Arch. 2001;442(3):329–335. doi: 10.1007/s004240100547. 11484762

[7] Kulkarni S, Parati G, Bangalore S, et al. Blood pressure variability: a review. J Hypertens. 2025;43(6):929–938. doi: 10.1097/hjh.0000000000003994. 40084481 PMC12052075

[8] Kang SH, Park SY, Lim YJ, et al. Impact of phosphate variability in patients undergoing hemodialysis. Nutrients. 2025;17(9):1528. doi: 10.3390/nu17091528. 40362836 PMC12073743

[9] Zhao S, Zhao Q, Xu Y, et al. The association between albumin corrected calcium levels and mortality in ICU patients undergoing maintenance hemodialysis. Sci Rep. 2025;15(1):12086. doi: 10.1038/s41598-025-96454-0. 40204886 PMC11982323

[10] Wang J, Lv M, Zach O, et al. Calcium–Polystyrene sulfonate decreases inter-dialytic hyperkalemia in patients undergoing maintenance hemodialysis: a prospective, randomized, crossover study. Ther Apher Dial. 2018;22(6):609–616. doi: 10.1111/1744-9987.12723. 30109784

[11] Marshall WR, Curran GA, Traynor JP, et al. Sodium zirconium cyclosilicate treatment and rates of emergency interventions for hyperkalaemia: a propensity–score weighted case–control study. Clin Kidney J. 2024;17(11):sfae313. doi: 10.1093/ckj/sfae313. 39669394 PMC11635375

[12] Vergili JM, Wolf RL. Nutrition practices of renal dietitians in hemodialysis centers throughout the united states: a descriptive study. J Ren Nutr. 2010;20(1):8.e1–8.e16. doi: 10.1053/j.jrn.2009.06.019. 19796966

[13] de Rooij ENM, Dekker FW, Le Cessie S, et al. Serum potassium and mortality risk in hemodialysis patients: a cohort study. Kidney Med. 2022;4(1):100379. doi: 10.1016/j.xkme.2021.08.013. 35072043 PMC8767120

[14] Karaboyas A, Zee J, Brunelli SM, et al. Dialysate potassium, serum potassium, mortality, and arrhythmia events in hemodialysis: results from the dialysis outcomes and practice patterns study (DOPPS). Am J Kidney Dis. 2017;69(2):266–277. doi: 10.1053/j.ajkd.2016.09.015. 27866964 PMC5520979

[15] Men R, Zhu M, Li P, et al. Associations between serum potassium variability and mortality in patients undergoing maintenance hemodialysis: a retrospective study. Sci Rep. 2024;14(1):29998. doi: 10.1038/s41598-024-80709-3. 39622893 PMC11611915

[16] Feng M, Li D, Hao F, et al. Association of serum potassium variability with 60-day mortality and cardiovascular events after COVID-19 infection in maintenance hemodialysis patients. Int J Med Sci. 2024;21(2):277–283. doi: 10.7150/ijms.88529. 38169716 PMC10758154

[17] Yamaguchi K, Kitamura M, Otsuka E, et al. Association between annual variability of potassium levels and prognosis in patients undergoing hemodialysis. Clin Exp Nephrol. 2023;27(10):873–881. doi: 10.1007/s10157-023-02368-4. 37318722

[18] Zhao X, Hou FF, Liang X, et al. High facility-level serum potassium variability associated with mortality in hemodialysis patients: results from Chinese Dialysis Outcomes and Practice Patterns Study (DOPPS). Ren Fail. 2023;45(1):2211157. doi: 10.1080/0886022x.2023.2211157. 37293774 PMC10259339

[19] Ohnishi T, Kimachi M, Fukuma S, et al. Postdialysis hypokalemia and all-cause mortality in patients undergoing maintenance hemodialysis. Clin J Am Soc Nephrol. 2019;14(6):873–881. doi: 10.2215/cjn.07950718. 31048327 PMC6556735

[20] Bignotto LH, Kallás ME, Djouki RJT, et al. Electrocardiographic findings in chronic hemodialysis patients. J Bras Nefrol. 2012;34(3):235–242. doi: 10.5935/0101-2800.20120004. 23099828

[21] Kim ED, Watt J, Tereshchenko LG, et al. Associations of serum and dialysate electrolytes with QT interval and prolongation in incident hemodialysis: the predictors of arrhythmic and cardiovascular risk in end-stage renal disease (PACE) study. BMC Nephrol. 2019;20(1):133. doi: 10.1186/s12882-019-1282-5. 30999887 PMC6474045

[22] Xu Q, Xu F, Fan L, et al. Serum potassium levels and its variability in incident peritoneal dialysis patients: associations with mortality. PLoS One. 2014;9(1):e86750. doi: 10.1371/journal.pone.0086750. 24475176 PMC3903570

[23] Wang G, Ma K, Ma Z, et al. Short-term blood pressure variability and outcomes in non-dialysis chronic kidney disease. Front Med (Lausanne). 2022;9:911205. doi: 10.3389/fmed.2022.911205. 36237550 PMC9550867

[24] Nakazato Y, Kurane R, Hirose S, et al. Variability of laboratory parameters is associated with frailty markers and predicts non-cardiac mortality in hemodialysis patients. Clin Exp Nephrol. 2015;19(6):1165–1178. doi: 10.1007/s10157-015-1108-0. 25788369

[25] Ohno Y. Role of systemic inflammatory response markers in urological malignancy. Int J Urol. 2019;26(1):31–47. doi: 10.1111/iju.13801. 30253448

[26] Stolarski AE, Wee K, Young L, et al. Application of creatinine height index in patients with trauma for the evaluation of psoas muscle mass: a clinical validation study. JPEN J Parenter Enteral Nutr. 2023;47(6):766–772. doi: 10.1002/jpen.2524. 37218671 PMC10602390

[27] Varvelo L, Lynd JK, Citty B, et al. Formally exact simulations of mesoscale exciton diffusion in a light-harvesting 2 antenna nanoarray. J Phys Chem Lett. 2023;14(12):3077–3083. doi: 10.1021/acs.jpclett.3c00086. 36947483 PMC10069740

[28] Skriver MV, Sandbæk A, Kristensen JK, et al. Relationship of HbA1c variability, absolute changes in HbA1c, and all-cause mortality in type 2 diabetes: a Danish population-based prospective observational study. BMJ Open Diabetes Res Care. 2015;3(1):e000060. doi: 10.1136/bmjdrc-2014-000060. PMC4317527 25664182

[29] Rothwell PM, Howard SC, Dolan E, et al. Prognostic significance of visit-to-visit variability, maximum systolic blood pressure, and episodic hypertension. Lancet. 2010;375(9718):895–905. doi: 10.1016/s0140-6736(10)60308-x. 20226988

[30] Hoppe LK, Muhlack DC, Koenig W, et al. Association of abnormal serum potassium levels with arrhythmias and cardiovascular mortality: a systematic review and meta-analysis of observational studies. Cardiovasc Drugs Ther. 2018;32(2):197–212. doi: 10.1007/s10557-018-6783-0. 29679302

[31] Pun PH. The interplay between CKD, sudden cardiac death, and ventricular arrhythmias. Adv Chronic Kidney Dis. 2014;21(6):480–488. doi: 10.1053/j.ackd.2014.06.007. 25443573 PMC4255332

[32] Iwagami M, Kanemura Y, Morita N, et al. Association of hyperkalemia and hypokalemia with patient characteristics and clinical outcomes in japanese hemodialysis (HD) patients. J Clin Med. 2023;12(6):2115. doi: 10.3390/jcm12062115. 36983118 PMC10058536

[33] Zhang H, Liu J, Liu X, et al. Pre- to post-dialysis potassium gradient and mortality in patients on hemodialysis: a propensity-matched analysis. Ther Apher Dial. 2024;29(2):189–199. doi: 10.1111/1744-9987.14213. 39342973

[34] Huo Z, Zhu X, Yang Y, et al. Association of hypokalemia with mortality in patients undergoing hemodialysis: a systematic review and meta-analysis. Semin Dial. 2025;38(2):85–101. doi: 10.1111/sdi.13234. 39658931

[35] Fishbane S, Jadoul M, Dember LM, et al. MO214 evaluation of the effect of a potassium binder on arrhythmia-related cardiovascular outcomes in patients on chronic haemodialysis with recurrent hyperkalaemia: design and rationale for the sodium zirconium cyclosilicate dialize-outcomes study. Nephrol Dials Transplant. 2021;36(Supplement_1). doi: 10.1093/ndt/gfab092.0092.

[36] Schüttler D, Schönermarck U, Wenner F, et al. Large potassium shifts during dialysis enhance cardiac repolarization instability. J Nephrol. 2021;34(4):1301–1305. doi: 10.1007/s40620-020-00880-4. 33058038 PMC8357640

[37] Weiss JN, Qu Z, Shivkumar K. Electrophysiology of hypokalemia and hyperkalemia. Circ Arrhythm Electrophysiol. 2017;10(3):e004667. doi: 10.1161/circep.116.004667. PMC5399982 28314851

[38] Selen T, Ulusal Okyay G, Ayerden Ebinç F, et al. Does control nutritional status (CONUT) score predict early and long-term mortality at the initiation of maintenance hemodialysis? Int J Gen Med. 2025;18:3775–3786. doi: 10.2147/ijgm.s533080. 40655306 PMC12254193

[39] Dawson J, Smyth B, Ryan M, et al. The impact of frailty and malnutrition on hospitalisation and survival in people with kidney failure. J Nephrol. 2025;38(8):2251–2259. doi: 10.1007/s40620-025-02356-9. 40699534 PMC12630193

[40] Wang C, Shen J, Charalambous C, et al. Modeling biomarker variability in joint analysis of longitudinal and time-to-event data. Biostatistics. 2024;25(2):577–596. doi: 10.1093/biostatistics/kxad009. 37230468 PMC11017116

[41] Levin A, Ahmed SB, Carrero JJ, et al. Executive summary of the KDIGO 2024 clinical practice guideline for the evaluation and management of chronic kidney disease: known knowns and known unknowns. Kidney Int. 2024;105(4):684–701. doi: 10.1016/j.kint.2023.10.016. 38519239

[42] Lew SQ, Ronco C. Use of eHealth and remote patient monitoring: a tool to support home dialysis patients, with an emphasis on peritoneal dialysis. Clin Kidney J. 2024;17(Suppl 1):i53–i61. doi: 10.1093/ckj/sfae081. 38846414 PMC11151118

[43] Nobakht E, Raru W, Dadgar S, et al. Precision dialysis: leveraging big data and artificial intelligence. Kidney Med. 2024;6(9):100868. doi: 10.1016/j.xkme.2024.100868. 39184285 PMC11342780

[44] Torshizi HM, Omidi N, Khorgami MR, et al. Artificial intelligence-based model for automatic real-time and noninvasive estimation of blood potassium levels in pediatric patients. Ann Pediatr Cardiol. 2024;17(2):116–123. doi: 10.4103/apc.apc_54_24. 39184121 PMC11343398

