# Supplementary material for: Dynamic potassium variability (K-CV) as an independent predictor of mortality in maintenance hemodialysis
Source: Ren Fail. 2026 Sep 6;48(1):2664912. doi: 10.1080/0886022X.2026.2664912 (PMC13552294; doi:10.1080/0886022X.2026.2664912)
Supplement: Supplementary file 1 [file IRNF_A_2664912_SM2656.docx]

#### Table S1. Schoenfeld residual test for proportional hazards assumption

| **Covariate** | **rho** | **χ²** | **P value** |
| --- | --- | --- | --- |
| **log(K-CV)** | **0.041** | **0.107** | **0.744** |
| **Age** | **0.059** | **0.257** | **0.612** |
| **Albumin** | **-0.031** | **0.082** | **0.775** |
| **Creatinine** | **-0.072** | **0.395** | **0.530** |
| **spKt/V** | **0.048** | **0.128** | **0.721** |
| **CRP** | **-0.066** | **0.341** | **0.559** |
| **Serum sodium** | **0.015** | **0.029** | **0.866** |
| **Serum phosphorus** | **-0.038** | **0.094** | **0.759** |
| **Potassium-sparing medication** | **-0.022** | **0.046** | **0.831** |
| **Global test** | **—** | **2.103** | **0.909** |

**Table S2. Comparison of Model Performance Before and After Inclusion of log(K-CV)**

| **Model** | **Variables included** | **Harrell’s C-index (95% CI)** | **AIC** | **ΔC-index** | **ΔAIC** |
| --- | --- | --- | --- | --- | --- |
| Model 1 (Baseline model) | Age, Albumin, Creatinine, spKt/V, CRP, Serum Sodium, Serum Phosphorus, Total Cholesterol | 0.701 (0.673–0.729) | 828.4 | – | – |
| Model 2 (Baseline + log(K-CV)) | All variables in Model 1 + log(K-CV) | 0.734 (0.705–0.763) | 817.2 | +0.033 | −11.2 |
